# Reply concerning: Correlation between acetabular index at 3 and 12 months of age: a longitudinal radiographic study of 228 neonates treated for 6 or 12 weeks with the von Rosen splint for developmental dysplasia of the hip

**DOI:** 10.2340/17453674.2026.45820

**Published:** 2026-04-16

**Authors:** Adam SAND, Daniel WENGER, Henrik DÜPPE, Carl Johan TIDERIUS

**Affiliations:** Orthopaedics, Department of Clinical Sciences Lund, Lund University, Lund, and Department of Orthopaedics, Skåne University Hospital, Lund, Sweden

Sir, — We thank Dr Kocak for his insightful comments [1] on our article “Correlation between acetabular index at 3 and 12 months of age: a longitudinal radiographic study of 228 neonates treated for 6 or 12 weeks with the von Rosen splint for developmental dysplasia of the hip” [2].

He put our main conclusion nicely in words when stating that “3-month AI should not be used in isolation to trigger additional abduction treatment.” We agree that the retrospective study design comes with inherent limitations, bilaterality can complicate matters, and most certainly there must be other factors than treatment duration that affect acetabular morphology and remodeling (or there would have been no variation between our study subjects).

The link between acetabular dysplasia in infants around 3 months of age and dysplasia in adulthood is obscure at best, if there even is any. In Graf’s original material, acetabular “dysplasia” before 3 months of age (type IIa hips) was present in 90% of sonographically examined children [3], whereas the prevalence of hip dysplasia (lateral center–edge angle ≤ 20°) in the adult Swedish population is 5.2% [4]. In our present study, already at 12 months age the correlation to radiographic appearance at 3 months in the same children was weak [2].

Although comparisons between hip morphology assessed with radiography versus sonography (Graf) must be made with caution, some questions are inevitably raised. We already see in the Swedish Pediatric Orthopedic Quality Register that treatment rates vary greatly within Sweden. Most pediatric orthopedic units in Sweden use hip instability as the indication for brace treatment, in uncertain cases verified with dynamic ultrasound [5]. However, one center uses acetabular morphology (assessed by Graf sonography) as an additional indication for treatment, which leads to much higher treatment rates [6]. The results of our study do not support such treatment. To put it in a larger perspective, currently in Sweden where developmental dysplasia of the hip (DDH) screening is predominantly focused on clinical examinations in neonates, the total rate of dislocations discovered later than 2 weeks of age is the same as the rate of open surgical reductions in Austria (where screening relies more heavily on sonographic morphology) [7,8]. Although universal clinical screening during the first week of life seems to give the lowest rate of late diagnosed hip dislocations in the published literature, it is dependent on skilled examiners on the maternity wards [9].

In line with Dr Kocak’s suggestions for future analyses, since 2023 the Swedish Pediatric Orthopedic Quality Register also includes early DDH (all brace treatments) and evaluation is performed by radiography at 18 months of age [6]. We hope to be able to shed more light on these important questions in the near future.


**Adam SAND, Daniel WENGER, Henrik DÜPPE, and Carl Johan TIDERIUS** *Orthopaedics, Department of Clinical Sciences Lund, Lund University, Lund, and Department of Orthopaedics, Skåne University Hospital, Lund, Sweden* *Correspondence: adam.sand@med.lu.se*

